# Evaluation of Different Concentrations of Graphene on the Structural and Optical Properties of Carboxymethyl Cellulose Sodium

**DOI:** 10.3390/polym17030391

**Published:** 2025-01-31

**Authors:** Nadiah Y. Aldaleeli, Mohamed Madani, Samera Ali Al-Gahtany, Hanan Elhaes, Rania Badry, Medhat A. Ibrahim

**Affiliations:** 1 Department of Physics, College of Science and Humanities-Jubail, Imam Abdulrahman Bin Faisal University, Jubail 35811, Saudi Arabia; 2 Department of Physics, Faculty of Science, University of Jeddah, Jeddah 21959, Saudi Arabia; saalkahtanee@uj.edu.sa; 3 Physics Department, Faculty of Women for Arts, Science and Education, Ain Shams University, Cairo 11757, Egypt; hanan.elhaes@women.asu.edu.eg (H.E.); rania.badri@women.asu.edu.eg (R.B.); 4 Spectroscopy Department, National Research Centre, 33 El-Bohouth St., Dokki, Giza 12622, Egypt; medahmed6@yahoo.com; 5 Molecular Modeling and Spectroscopy Laboratory, Centre of Excellence for Advanced Science, National Research Centre, 33 El-Bohouth St., Dokki, Giza 12622, Egypt

**Keywords:** food packaging, CMC/G nanocomposites, SEM, FT-IR, optical analysis, DFT

## Abstract

Developing sustainable and green packaging products that protect foods and preserve their unique properties from UV radiation, which causes photochemical damage, is one of the extensive challenges in the food-packaging industry. Accordingly, carboxymethyl cellulose sodium (CMC)/graphene (G) nanocomposites that contained different weight percentages were prepared by a mechanical milling method. The influence of the G on the chemical composition and optical properties of the nanocomposites were studied by different techniques. SEM and FT-IR analyses confirmed the interaction between the CMC and G. The XRD spectrum showed that the crystallite size of the CMC decreased with G addition. The findings showed that changing the G concentration modified the CMC’s optical properties. The CMC’s transmittance decreased to 52%, 49%, and 57% in the UV-C (200–280), UV-B (280–320 nm), and UV-A (320–400) regions, respectively, with the addition of 2 wt.% of G. Moreover, the optical band gap decreased to 4.80 eV, while the Urbach energy increased from 0.34 to 0.94 eV as the G content increased. The density functional theory (DFT) assumption was followed to establish the electronic properties and vibrational spectrum of the CMC/G model. The theoretically determined IR and experimental FT-IR spectra of the CMC/G nanocomposites showed good agreement. The obtained results show that these nanocomposites are good candidates for food packaging.

## 1. Introduction

Because of their durability and adaptability, synthetic polymers are now utilized in an extensive range of industries, including packaging, opto-electronics, textiles, construction, and auto parts [1,2]. The majority of these polymers are non-biodegradable, non-renewable, and degrade over a long time. The waste of these polymers contributes to the expansion of landfills and endangers the health of the environment due to society’s fast consumption. Biopolymers made from natural sources show promise as remedies for this problem. Proteins, carbohydrates, and lipids are examples of natural and renewable resources that can be converted into biodegradable biopolymers [3,4,5]. These polymers are thought to be more environmentally friendly than non-biodegradable polymers because they can naturally decompose into smaller molecules [6]. Biodegradable biopolymers include cellulose, gelatin, chitosan, and alginate. Plant cell walls naturally contain cellulose, the most common organic polymer on the planet. Numerous plant sources, including cellulose, wood, cotton, and maize stalks, can provide it. Papermaking, textiles, and food production are just a few of the many uses for cellulose [7,8,9,10].

Carboxymethyl cellulose sodium (CMC) is a polymer that is soluble in water and produced from cellulose. The carboxymethylation process, in which carboxymethyl groups are replaced by OH groups in the cellulose chains, is the basis for the manufacture of CMC. CMC is a semi-crystalline, semi-synthetic biopolymer that is inexpensive, non-toxic, and has good gelling and film-forming properties. Nevertheless, its poor mechanical properties and electrical conductivity do not meet the requirements for practical applications [11,12,13].

Materials known as biopolymer nanocomposites are created by mixing biopolymers with nanoscale fillers. Because of their special physical and chemical characteristics, carbon nanofillers, like graphenes and carbon nanotubes, can be used in a comprehensive range of fields, such as food packaging, electronics, energy storage, and medicine [12,14,15]. The material gains enhanced properties, like a low band gap energy, a high elastic modulus, high conductivity, and thermal stability, when nanofillers are evenly distributed throughout the biopolymer matrix [9,16,17,18]. The final characteristics of a biopolymer nanocomposite are greatly influenced by a number of variables, including the intrinsic characteristics of the used filler (carbon), its contents, grading, size, dispersion throughout the polymer chains, and compatibility with the polymer [15].

In the manufacturing of polymer composites, graphene (G) is an affordable substitute for carbon nanotubes (CNTs) as fillers [14]. Therefore, the research community is very interested in developing G-polymer composites suitable for commercial use that have properties similar to those of CNT–polymer composites. This study offers a crucial viewpoint for the development of new compositions with enhanced operating conditions [19].

In semiconductor (SC) physics, the energy necessary for an electron to move from the valence band (VB) to the conduction band (CB) is called the “band gap energy”. This energy is obtained from the difference in energy between the upper VB and the lower CB of the SC material. Band gap (BG) engineering, or band gap energy control, is a fundamental component of photonic and optoelectronic products using semiconductive polymer compounds. In a previous study, we demonstrated that the BG energy of the resulting composites can be effectively altered by adding carbon fillers, such as graphene and CNT, to the nonconductive polymers [20,21,22,23,24]. For certain applications, the BG energy can be precisely adapted by changing the concentration of these fillers in the composite.

Optical absorbance spectroscopy is the most popular method for describing the physical characteristics of polymer composites. For predicting a polymer composite’s performance and suitability for a variety of applications, including optical filters, food packaging, photoluminescence, lasers, photovoltaics, solar cells, water splitting, photocatalysis, sensors, switchable membranes, and diodes, it is especially crucial to determine its optical band gap energy [25,26,27,28]. This method is an invaluable resource for researchers in many scientific fields because it is easy to use and yields accurate results.

As stated in the above literature regarding the ever-evolving landscape of the food industry, the role of CMC stands out as an essential and versatile component. Therefore, it is important to study it based on density functional theory (DFT), together with spectroscopic analyses, to better understand its electronic properties. Furthermore, studies that examined the optical characteristics of CMC/G nanocomposites are uncommon in the literature. In this study, we prepared CMC/G nanocomposite pellets with different concentrations of G using a simple and eco-friendly mechanical milling technique. Using the Tauc model, we methodically investigated how the incorporation of G affected the optical transmittance and band gap energy (Eg) of the CMC nanocomposites. The morphology and crystallite size was determined using SEM and XRD. With the B3LYP/6-31G (d, p) quantum mechanical model, some important physical parameters were calculated for the CMC and CMC/G, such as the total dipole moment (TDM), HOMO/LUMO band gap energy, molecular electrostatic potential (MESP), and IR spectrum. DFT was used to evaluate the interaction mechanism between the CMC and G.

## 2. Materials and Methods

### 2.1. Materials

CMC was acquired from K. Patel Chemo-pharma PVT, India, and had a molecular weight of 2.5 × 10^5^ g/mol. Sigma-Aldrich, St. Louis, MO, USA, supplied the graphene NPs (Aldrich 900413). The common solvent for both the CMC and G was distilled water (D.W.). After thoroughly cleaning the glassware with a soap solution, D.W. was used to wash it.

### 2.2. CMC/GO Nanocomposites Syntheses

The milling technique was used to prepare the CMC/G nanocomposites (NCs). A total of 1 g of CMC and different weight percentages of G (0.2 wt.%, 0.4 wt.%, 0.8 wt.%, 1 wt.%, 2 wt.%, and 3 wt.%) were inserted in an agitator mortar. Then, the obtained composition was grounded for ten minutes to obtain CMC/G NCs.

### 2.3. Characterization Techniques

The surface morphology of the CMC nanocomposites was evaluated using a Hitachi S-4800 (Tokyo, Japan) scanning electron microscope (SEM). The operating voltage during the measurements was 20 kV. After this, the particles were gathered on carbon-coated copper grids. The structure of the CMC nanocomposites was examined using Fourier transform infrared spectroscopy (FTIR, vertex 70, Bruker, Ettlingen, Germany) in the 4000–400 cm^−1^ range. When using a diamond crystal (type II alpha), the diamond ATR accessory could penetrate a depth of 2 µm. Each spectrum underwent a total of 40 scans. The background due to air was tested at a resolution of 4 cm^−1^ using the same parameters. An X-ray diffractometer (XRD) fitted with Cu Kα radiation from Rigaku Smart LabTM (Tokyo, Japan) was used to analyze the CMC nanocomposites. The optical properties were examined at room temperature using a UV–Vis spectrophotometer (V-570 UV/VIS/NIR, JASCO, Tokyo, Japan) in the wavelength range of 200–800 nm. The samples were pre-heated at 25 °C before testing. Prior to the analysis, the synthesized nanocomposites were sonicated for 30 min with 30 s/30 s on/off cycles using a Sonics Vibracell (Newtown, CT, USA) ultrasonic processor (500 W, 20 kHz). The spectrum of the sample was measured and the optical background was deducted.

### 2.4. Calculation Details

The Gaussian 09 program at the Molecular Modelling and Spectroscopy Laboratory, Centre of Excellence for Advanced Science, National Research Centre, was used to study the mechanism of interaction between the CMC and G [29]. GaussView 5.0 was used to arrange the input files according to the density functional theory (DFT) [30]. The Becke, 3-parameter, Lee–Yang–Parr (B3LYP) hybrid functional was used to optimize the studied structure because it was shown to accurately represent the electronic structure of carbon-based nanosystems. To balance the accuracy and computational efficiency, the CMC/G model molecule was fully optimized in the gas phase using the B3LYP/6-31G (d, p) model. The B3LYP mode is an integration of the Lee–Yang–Parr correlation mode (LYP) and Beck’s three-parameter hybrid exchange mode (B3). The polar bonding in molecules was more accurately described using the 6-31G (d, p) basis set with “d” polarization functions for the heavy atoms and “p” polarization functions for the hydrogen atoms [31,32,33].

At the B3LYP/6-31G (d, p) level, the total dipole moment (TDM), HOMO/LUMO band gap energy, molecular electrostatic potential (MESP), and theoretical IR spectrum were calculated. The stability of the optimized geometries was shown in a frequency study that used the same theoretical level. The improved geometry at the real and positive minima in the potential energy surface is highlighted by the absence of negative eigenvalues across all the estimated frequencies [34].

## 3. Results

### 3.1. SEM Analysis

**Figure 1:**
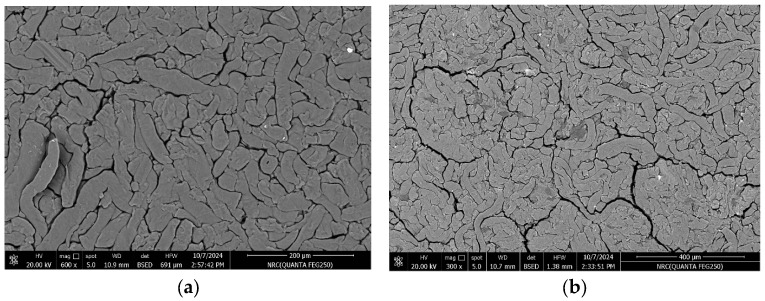

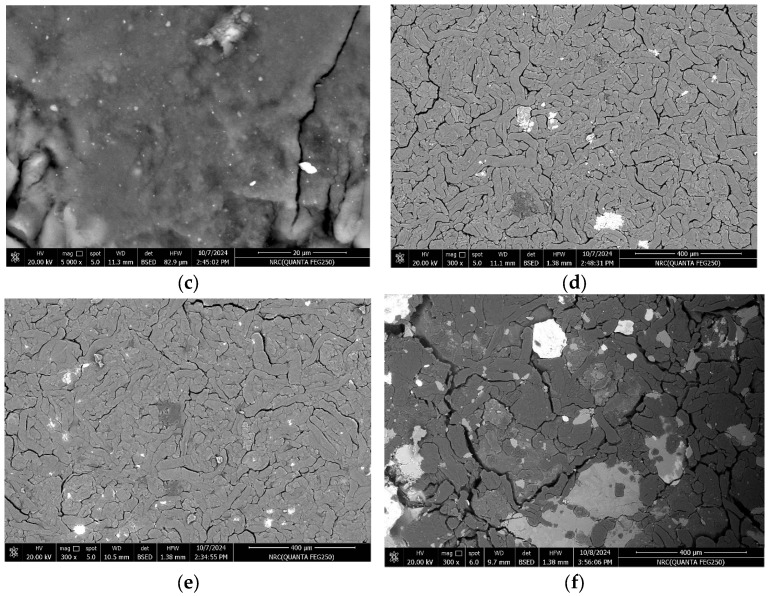
SEM images of (**a**) CMC (scale: 400 µm), (**b**) CMC/0.4% G (scale: 20 µm), (**c**) CMC/0.8% G (scale: 400 µm), (**d**) CMC/1% G (scale: 400 µm), (**e**) CMC/2% G (scale: 400 µm), and (**f**) CMC/3% G (scale: 400 µm).

The SEM images were a good indicator of the possible dispersion of G-incorporated CMC chains, as well as the interaction between the G and the polymer chains. The pictures indicate how the G affected the overall morphology and structure of the CMC. Thus, one could reasonably understand the properties of the CMC chain and its interaction with the G in all the obtained SEM images. SEM was used to evaluate the surface morphology of the CMC and CMC/G samples. The morphology of the investigated pure CMC (Figure 1a) was noted to be a rough layered surface. The results indicate that the nature of the CMC could be described as semi-crystalline with a flat texture. The SEM images of the CMC/GO nanocomposite sample (Figure 1b–f) show that the roughness increased with the addition of the visible spherical G nanoparticles. The images show that the G nanoparticles were well distributed and had uniform consistency in the CMC/GO matrix. However, some agglomerations and larger particle sizes of G nanoparticles could be seen in the CMC/GO composite. The observed roughness of the samples indicates the interaction of the G nanoparticles with different groups of the CMC. From the results, it could be concluded that the G particles were well and uniformly dispersed throughout the CMC chains, with a higher dispersion strength and less phase separation. This may be an indication of the superior quality of the CMC/G nanocomposite.

### 3.2. FT-IR Analysis

**Figure 2:**
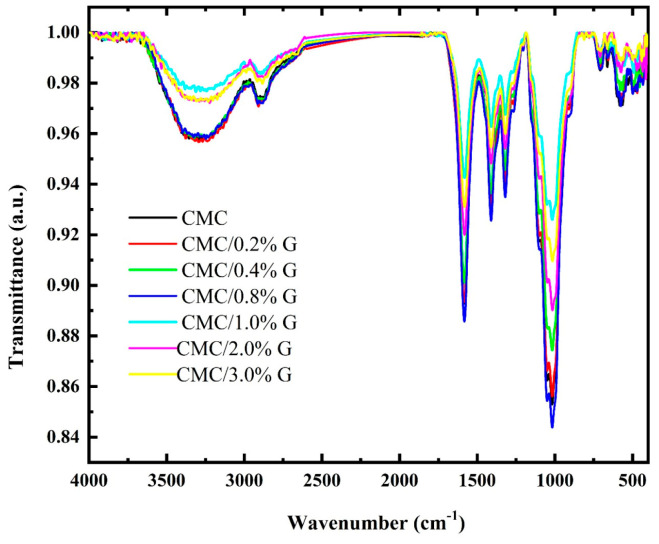
FTIR transmittance spectra of non-filled CMC and CMC nanocomposites.

To analyze the chemical structure, molecular features, and functional groups, FT-IR spectroscopy was performed for both the CMC and CMC/G. The FT-IR transmittance spectrum of the non-filled CMC and CMC/G NCs are shown in Figure 2. This figure displays the most distinctive bands of the pure CMC’s FT-IR spectrum. The large band at 3292 cm^−1^ in the typical CMC IR spectra indicates OH stretching vibration, while the bands around 2906 and 2850 cm^−1^ indicate symmetric and asymmetric stretching of the CH_2_ vibration [35,36,37]. The carboxylate (COO–) groups’ asymmetric and symmetric stretching vibrational modes are linked to bands at about 1584 cm^−1^ and 1411 cm^−1^, respectively [7].

Additionally, the bands at around 1319 and 1049 cm^−1^ were ascribed to the stretching of the bending vibration of –OH and the CH–O–CH_2_ group, respectively [16,17]. Table 1 presents the band assignments of the CMC and CMC nanocomposites. The resulting nanocomposites’ IR spectra showed a significant change in the IR intensity of the CMC’s characteristic bands. Additionally, the characteristic bands of the OH stretching and the CH_2_ symmetric and asymmetric stretching vibration underwent a strong shift due to the addition of graphene, suggesting that the CMC and G interacted via the formation of hydrogen bonding. On the other hand, this shift in the peak position indicates a robust hydrogen bonding interaction between the CMC and G. However, the CMC got wider when the G was introduced; this suggests that the CMC and G were compatible. The overall conclusion from Figure 2 is that the G formed a composite with the CMC for the different studied G concentrations. Furthermore, the FTIR spectrum of the CMC/G nanocomposite contained an abundance of functional groups, all of which had excellent chemical activity, which demonstrated the mutual enhancement between the CMC and G.

### 3.3. XRD Analysis

**Figure 3:**
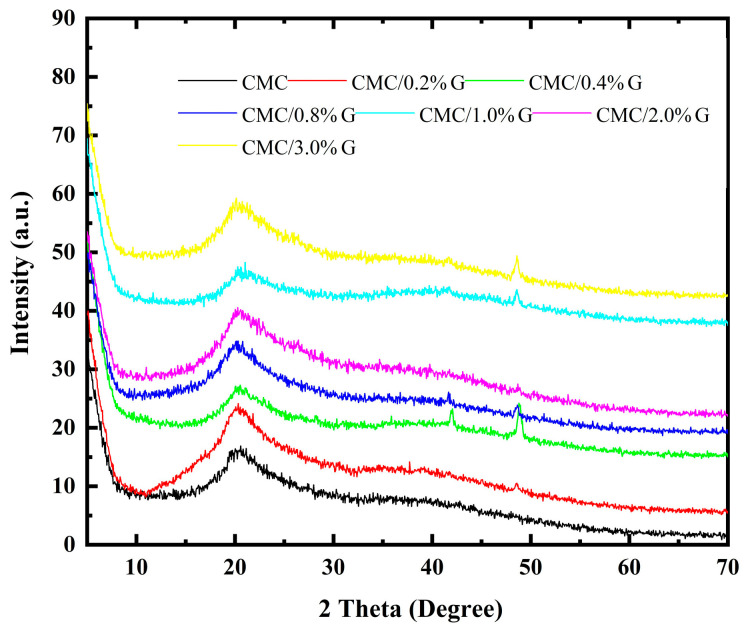
XRD chart of non-filled CMC and CMC/G nanocomposites.

The X-ray diffraction technique was used to analyze the phase composition and crystallinity of the prepared CMC and CMC/G nanocomposite samples. Figure 3 exhibits the pure CMC and CMC-doped XRD patterns with 0.2 wt.%, 0.4 wt.%, 0.8 wt.%, 1 wt.%, 2 wt.%, and 3 wt.% G. This figure shows a broad diffraction peak of pure CMC at nearly 20°, which reflected its amorphous nature.

The CMC XRD peak profiles slightly changed due to the addition of the different weight percentages of G. This indicates that the degree of crystallinity of CMC was affected by the addition of nanofiller. This finding lends credence to the theory that changes in the electrostatic reaction of the functional groups of CMC and the G sheet cause variations in the CMC structure. New peak centers at 48.58° were observed when 0.2 wt.% of G was added. The (100) reflecting plane of G was represented by this diffraction peak [38]. Increasing the concentration of G to 0.4 wt.%, 0.8 wt.%, 1 wt.%, 2 wt.%, and 3 wt.% resulted in a new diffraction peak at 41.93°. Furthermore, increasing the G concentration resulted in a shift in the characteristic XRD peak of G.

The crystallite size of the CMC nanocomposites was calculated using the Scherrer formula:

(1) $$D=\frac{K\lambda }{\beta Cos\theta }$$

In this instance, “D” (Å) is the average size, and K (=0.93) is the shape factor for spherical particles. The full width at half maximum is β, and the angle of the XRD is θ. The wavelength λ of the Cukα X-rays tube was 1.5406 Å. The value of θ is obtained by taking half of the 2θ. The crystal size is calculated by combining these values with the other numerical values included in Equation (1). The determined crystal sizes of the CMC and CMC nanocomposite samples (Table 2) were 80.71, 28.48, 34.76, 41.71, 42.93, 41.53, and 40.92 nm for the CMC doped with 0.2 wt.%, 0.4 wt.%, 0.8 wt.%, 1 wt.%, 2 wt.%, and 3 wt.% G, respectively. It was found that the crystallite size of CMC decreased with the addition of G, which confirmed the reduction in the optical BG value. Moreover, the increase in the crystallite size with increased graphene content from 0.4 wt.% to 2 wt.% may have been due to the agglomeration of the G NPs, which agreed with the obtained SEM results. At these concentrations (0.4 wt.% to 2 wt.%), G acted as an efficient nucleating agent to promote crystallization in the CMC by facilitating intrachain conformational ordering [39].

The XRD crystallite size results indicate that the incorporation of the G in the CMC significantly affected its crystallinity and overall properties. In line with the reported results, the interaction between the G and CMC primarily enhanced the polymer’s structural integrity and thermal stability [40].

### 3.4. Optical Analysis

#### 3.4.1. Optical Absorption

**Figure 4:**
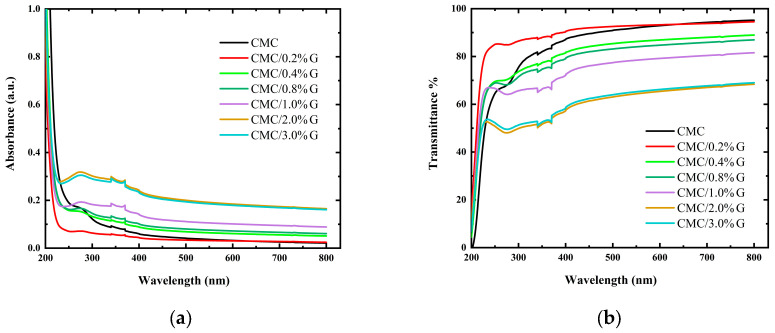

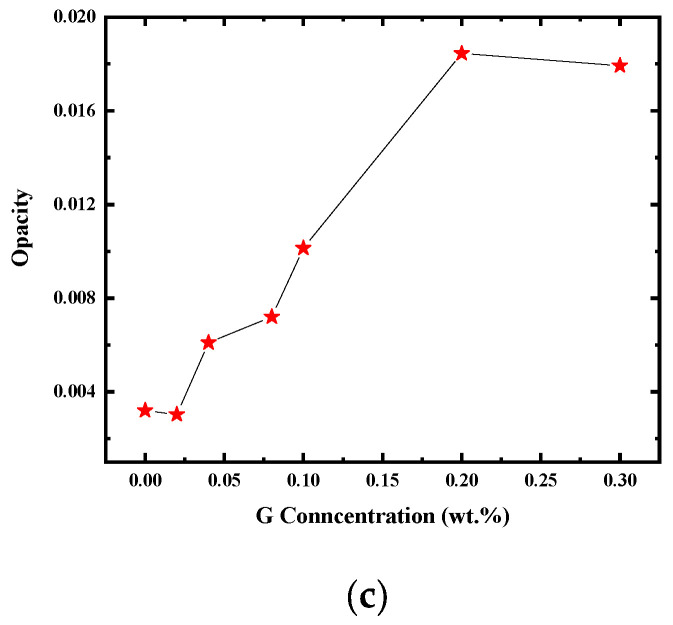
(**a**) UV–Vis absorbance, (**b**) transmittance behavior of non-filled CMC and CMC doped with different concentrations of G, and (**c**) variation in opacity with graphene concentration.

As technology advances, optical factors play a significant role in creating novel polymer composite materials that differ from conventional materials. The amount of filler added, in terms of the size, shape, and compatibility with the polymer, is the main factor that affects the properties of the material being prepared [41]. The intended optical characteristics can be somewhat altered by varying these parameters. UV–Vis spectroscopy analysis is the most useful technique for analyzing the optical characteristics of polymer composite materials. It provides insightful and useful information on the optical characteristics of the polymer and the filler. Figure 4a displays the absorption spectra, while Figure 4b displays the transmittance percentage of the non-filled CMC and filled CMC/G NCs in the 200–800 nm wavelength range. The semi-crystalline character of the CMC is demonstrated by the low absorption peak at 270 nm and the absorption shoulder at 235 nm in the absorption spectra of the pure CMC film shown in Figure 4a. The π → π* and n → π* transitions were responsible for these observed absorption bands [17,42]. It is noted that pure CMC film absorbs light in the ultraviolet and visible regions at wavelengths within or above 350 nm due to its relatively high transmittance [43]. The absorbance values in the nanocomposites significantly increased when the G concentration rose, which was correlated with an increase in the free charge carriers’ density in the latex. Additionally, the band peak that was visible at 263 nm became more noticeable. Additionally, increasing the concentration of G in the nanocomposites resulted in a new peak at nearly 350 nm corresponded to the C=C transition of G. The low intensity of the G NP’s typical peaks was due to its low contents in the obtained samples. These findings verify the existence of G NPs in the polymer matrix.

More detailed insight into Figure 4c, which presents the UV photon transmittance of non-filled CMC and CMC/G nanocomposites, can be indicated. The UV–Vis indicated a drop in the CMC/G nanocomposite starting from 270 nm as compared with the pure CMC. This could be an indication of blocking lower wavenumbers, and hence, the prevention of these wavelengths. The transmittance of the CMC/G nanocomposite was observed at about 270 nm to reach near-zero values in the UV-C range (100–280 nm). This indicates that the studied material acted as a protective layer against UV-C radiation. This finding is in good agreement with that stated recently in [44]. This paves the way toward quantifying the UV-shielding performance, as in the following section.

#### 3.4.2. UV-Shielding Performance

The BG structure and optoelectronic properties of polymers are studied and understood by the measurements of absorption and optical transmittance spectra of ultraviolet, visible, and near infrared radiation. As seen in Figure 4b, the optical transmittance significantly decreased with the addition of G NPs. This suggests that CMC and the G NPs interacted significantly. Two regions made up the transmittance spectrum: a strong absorption region in the UV spectra, where the absorption coefficient caused a significant decrease in transmittance, and a transparent region with low absorption in the visible region.

The pure CMC blocked 13–20% of UV radiation in the UV-A region (320–400 nm) and had an optical transmittance of about 92% in the UV–Vis region. The transmittance of incident photons involved those with shorter wavelengths. This was due the layer created by the intermolecular hydrogen interaction between the G NPs and the pure CMC matrix. Table 3 presents the transmittance values in the UV-C (200–280 nm), UV-B (280–320 nm), and UV-A regions for the pristine CMC and CMC/G NCs.

The transmittances of the CMC in the UV-B region decreased to 73, 71, 65, 49, and 51% due to the additions of 0.04 wt.%, 0.08 wt.%, 0.1 wt.%, 0.2 wt.%, and 0.3 wt.%, respectively. However, when 0.02 wt.% of G was added, the transmittance increased to 86% in the UV-B region, where this was due to the immiscibility between the CMC and G in this sample. In the UV-A region, the CMC/G nanocomposites transmitted only 81, 79, 72, 57, and 58% for CMC doped with 0.04 wt.%, 0.08 wt.%, 0.1 wt.%, 0.2 wt.%, and 0.3 wt.%, respectively.

Table 3 presents the advantages of the prepared nanocomposite samples in comparison with the previously reported data. In a previous work [16,17], the incorporation of 8 wt.% CuO NPs into CMC prevented 99% of UV-B and 99% of UV-A radiation. Meanwhile, the addition of 4 wt.% CuO@ZnO core/shell NPs into CMC blocked all the UV-C, UV-B, and UV-A rays. Accordingly, our findings make G-filled CMC nanocomposite samples attractive options for a range of optical and storage uses, such as UV shielding. These results were compared with a previous work that discovered that lignin enhances UV protection while reducing the transparency in cellulose-based films, as presented in Table 3 [45]. Moreover, the addition of 5% dopamine to PVA reduced the transmittance of PVA to 50%. However, in this work, the addition of 0.2% of G reduced the transmittance to 57%, which is a very good result.

The opacity versus the amount of G for the prepared composites, CMC NCs and pristine CMC, is displayed in Figure 4c at a wavelength of 600 nm. The following Equation (2) was used to calculate the opacity values for the prepared samples [13]:

(2) $$Opacity=\frac{A_{600}}{d}$$

where d and A_600_ are the thickness of the film (mm) and the absorbance at 600 nm, respectively. When the opacity value is high, it indicates that the transparency value of the composite is low. As presented in the figure, the opacity of CMC increased with the increasing G concentration.

#### 3.4.3. UV–Vis Absorption Parameters

Comparing the absorption values of CMC and its nanocomposite samples with varying thicknesses can be deceptive. This requires removing the sample thickness changes in order to obtain the unit length absorption values. Thus, for the optical analysis, 0.001 g of the nanocomposites was dissolved in 15 mL distilled water and then stirred for 1 h using a magnetic stirrer. Furthermore, the absorption coefficient provides crucial details regarding the electron transition and band structure. The following formula was used to determine the absorption coefficient (α), where A is the absorbance value [16]:

(3) $$\alpha =2.303\;\frac{A}{d}$$

**Figure 5:**
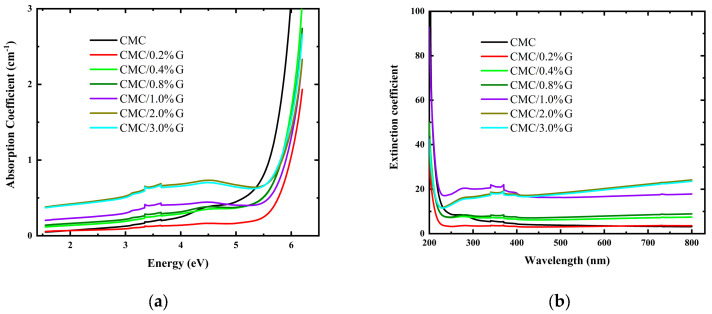
Variation in (**a**) absorption coefficient with energy and (**b**) extinction coefficient with wavelength for pure CMC and CMC doped with different concentrations of G.

The variable d stands for the sample thickness (d = 1 cm for the liquid samples). Figure 5a shows how the α of the pristine CMC and CMC/G nanocomposites varied with the wavelength using Equation (3). The absorbance variation and the absorption coefficient’s wavelength-dependent variation were comparable. It is evident from Figure 5a that both the energy and the G concentration affected the absorption coefficient. The likelihood of an electronic transition rose with an increase in the absorption coefficient in the high-energy region (Figure 5a). There was no chance of an electronic transition since the change in α diminished with lower energy. Table 4 shows the determined absorption edge values of the CMC due to the incorporation of the G NPs. Additionally, Figure 5a shows that some samples possessed two absorption shoulders as a result of the G addition.

When choosing materials for optoelectronic applications, the extinction coefficient k, which is the imaginary component of the complex refractive index, is a crucial factor. The following equation gives k, which stands for the percentage of electromagnetic energy lost as a result of scattering and absorption:

(4) $$k=\frac{\alpha \lambda }{4\pi }$$

Figure 5b displays the wavelength-dependent change in the extinction coefficient values computed using Equation (4) of the pure CMC and CMC/G NCs. The calculated α rose as the G concentration in the nanocomposites increased due to the increase in the free charged particles density. The extinction coefficient (EXC) values rose because of the photon energy loss that followed [10]. The scattering of the incident photons was the cause of the increase in the EXC values with increasing wavelength [47]. Consequently, the CMC/G nanocomposites’ energy reduction capabilities increased.

#### 3.4.4. Optical Band Gap Energy Calculations

As mentioned before, the energy required for an electron to move between the valence and conduction bands is the band gap, or Eg. Since it establishes the electrical and optical behavior of an SC substance, Eg is a strongly significant parameter in solid state physics. Determining the value of Eg precisely is essential for optimizing novel polymer composites with tailored properties for photonic and optoelectronic products. Numerous experimental methods, including Tauc, Kubelka–Munk (K-M), absorbance line fitting (ASF), McLean, Cody, inverse logarithmic derivative (ILD), and derivative approaches, can be used to determine a material’s Eg [48,49,50,51,52,53].

The Tauc model is commonly used to calculate the Eg value from the absorbed or transmitted ultraviolet light. The Tauc method is frequently used to find the Eg of a variety of polymer compositions, such as insulators and semiconductors. It is a straightforward and trustworthy technique that yields the precise approximations of a material’s optical band gap.

**Figure 6:**
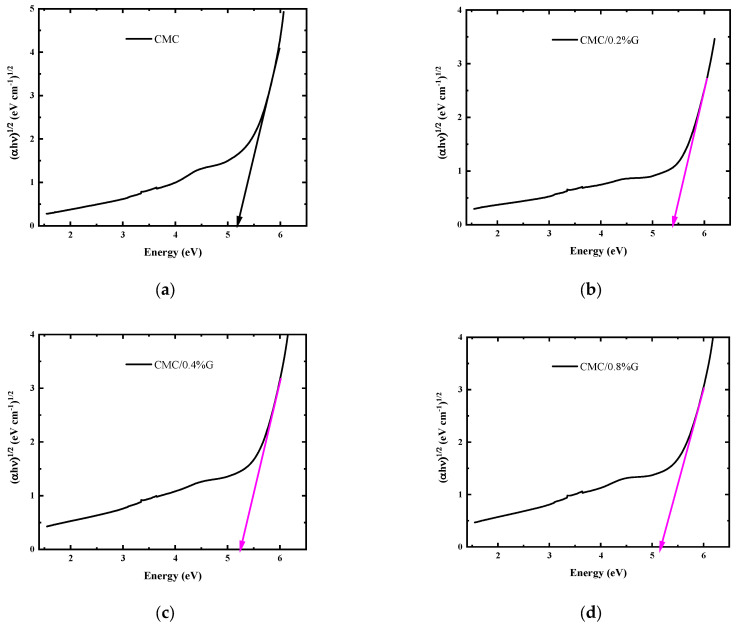

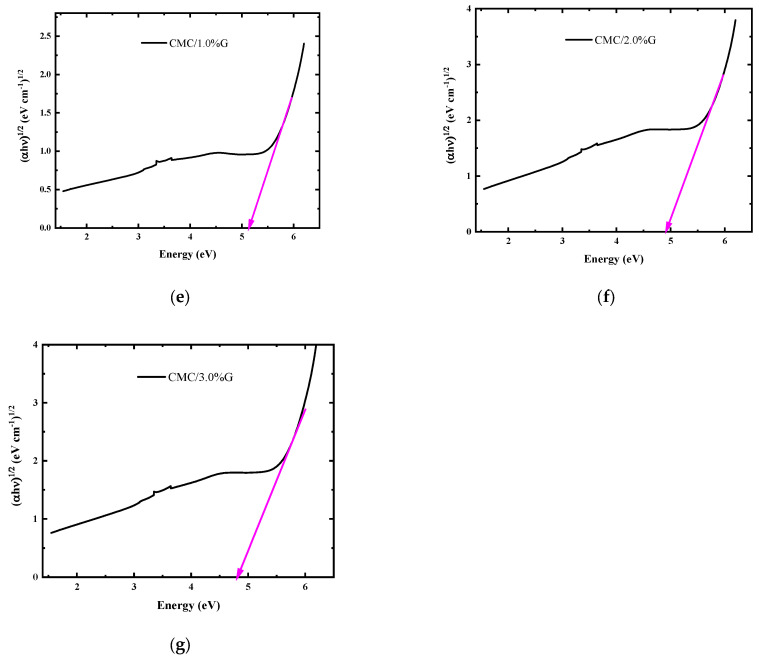
(αhν)^1/2^ versus (hν) plot of (**a**) pure CMC, (**b**) CMC/0.2% G NCs, (**c**) CMC/0.4% G NCs, (**d**) CMC/0.8% G NCs, (**e**) CMC/1.0% G NCs, (**f**) CMC/2.0% G NCs, and (**g**) CMC/3.0% G NCs.

According to the Tauc model [17], Eg is calculated from the following formula:

(αhv) = B(hν − Eg)^r^ (5)

where r is the Tauc exponent, B is the proportionality constant, and hv is the energy of the photons absorbed. The values of r = 1/2 (for a direct allowed transition), 2 (for an indirect allowed transition), 3/2 (for a direct forbidden transition), and 3 (for an indirect forbidden transition), depending on the type of electron transitions. According to our earlier research, the electrons in the CMC’s band gap [16] undergo an indirect transition from the valence to the conduction bands. In order to ascertain the indirect allowed transition, the photon energy is plotted against (αhν)^1/2^ in Figure 6. The indirect energies of Eg that were investigated by extrapolating the linear part of the obtained data to the hν-axis are shown in Table 4. The $E_{g}^{in}$ values of the CMC/G nanocomposites decreased from 5.27 to 4.81 eV with the increasing G content.

The incorporation of graphene nanoparticles into the CMC chain may lead to the production of energy levels within the CMC band gap, which leads to a decrease in the distance between the two ends of Eg. This, of course, leads to an improvement in the optical properties of the compound, and thus, an increase in the number of lattice defects. In addition, the enhancement in the optical property can be described by the increase in the degree of disordering due to the localized states produced within the CMC band gap [17].

#### 3.4.5. Determination of Urbach Energy

**Figure 7:**
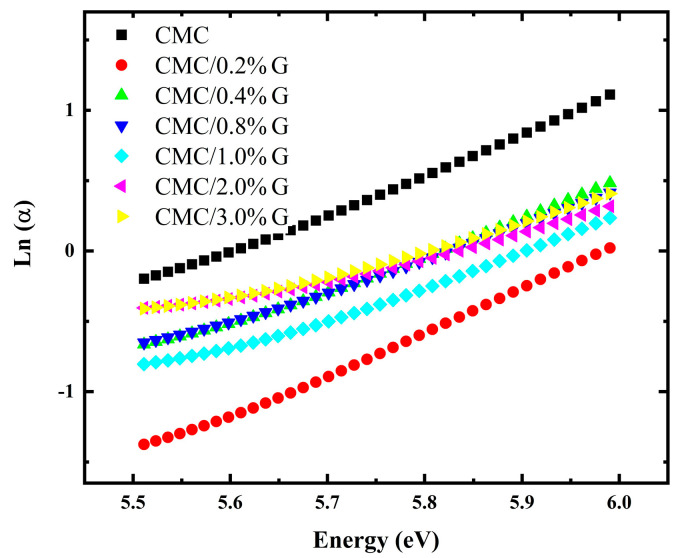
ln α vs. hν plots of pure CMC and CMC/G NCs.

An exponential tail known as the Urbach tail is present at the band edge of the absorption/absorption coefficient curve in amorphous, disordered, and low-crystalline materials [54]. This exponential tail distinguishes the understanding the transportation mechanisms of electrons in composite materials [55]. Following doping, the Urbach energy (Eu), which is a measure of the width of the band tail energy of localized states, indicates the degree of defect in Eg located between the VB and CB [17]. Urbach presented an empirical relationship that combines the absorption coefficient value of amorphous polymers versus the energy of the incident radiation at low energy values:

(6) $$\alpha =\alpha o\;e^{\left(\frac{E_{U}}{h\upsilon }\right)}$$

where Eu denotes the Urbach energy that correlates with the width of the band tails of localized states in the band gap, and αo is the composite’s material-specific parameter. The Urbach energy was determined using Equation (6), where the Eu value is given by the inverse slope of the line tangent in the lnα versus hv graphs. For the CMC/G nanocomposites, Figure 7 illustrates the lnα values versus hν of the prepared films. The slope of the linear portion in Figure 7 was used to obtain the Eu values shown in Table 4.

According to the Tauc model, the Eu values were found to change from 0.34 eV for pure CMC to 0.94 eV for the CMC NCs film containing 0.3 wt.% G. Furthermore, many studies on CMC-filled composites have shown that while the Urbach energy values rose with increased filling amounts, the band gap energy values decreased [16,17,56]. This finding suggests that by creating structural disorder, the incorporation of G into the CMC chains increased the amorphousness of CMC. The existence of sub-band states brought about by the creation of localized states between VB and CB was confirmed by this disorder. As a result, the decrease in Eg values was of great importance, as it could be observed that the Eg values decreased with increasing Eu values.

### 3.5. Interaction Mechanisms Between CMC and G

**Figure 8:**
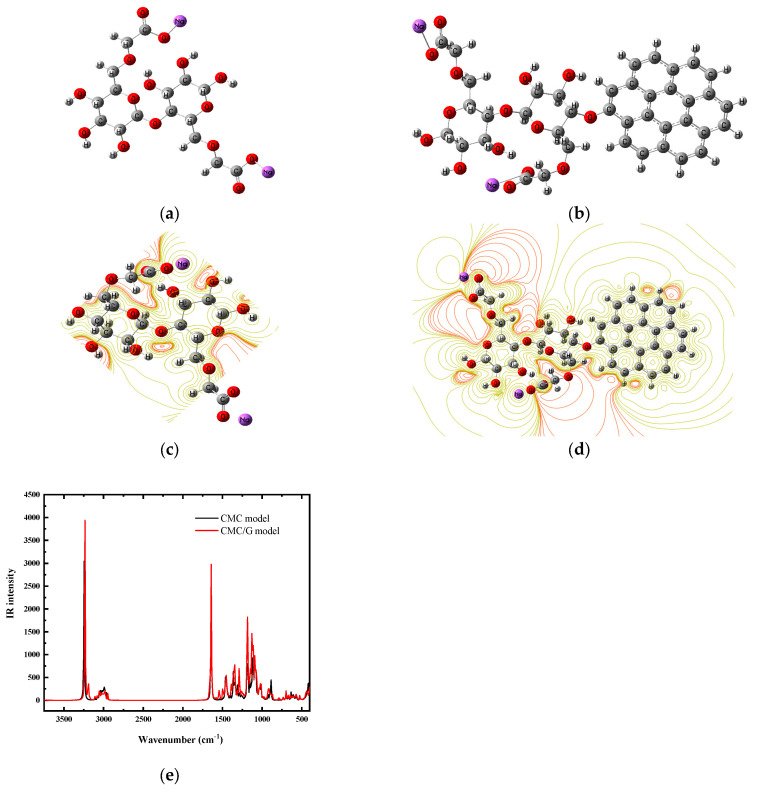
B3LYP/6-31G (d, p) calculated model molecules for the (**a**) **CMC** and (**b**) **CMC/G**; molecular electrostatic potential MESP for the (**c**) **CMC** and (**d**) **CMC/G**, and (**e**) the calculated IR frequencies for the CMC and CMC/G respectively.

Model molecules that represented the CMC and its interlinkage with the G were investigated to verify the experimental findings from the FT-IR and Eg analyses and to explain the mechanism of interaction between the CMC and G. A model of CMC/G that consisted of a single CMC unit that interacted with a 24-carbon G sheet was investigated. Previous studies showed that the oxygen atoms of the hydroxyl groups in CMC primarily interacted with the H atom of G [18,20,24]. Therefore, as illustrated in Figure 8a, we investigated one interaction site. The interaction between the CMC and G is presented in Figure 8b. Although the interaction through the OH was confirmed earlier [18,20,24], another confirmation in this work was conducted by calculating the IR of the CMC/G and comparing it with the experimental FTIR.

Suitable theoretical approaches for studying the electronic properties and reactivity of different nanocomposites are the total dipole moment (TDM) and the HOMO/UMO (the lowest unoccupied molecular orbital (LUMO) and highest occupied molecular orbital (HOMO) band gap energy (∆E), as tabulated in Table 5. Although the TDM of the CMC was calculated to be as high as 12.15 Debye, it slightly decreased to 8.05 Debye but was still high. Meanwhile, the HOMO/UMO band gap decreased from 4.22 eV to 3.68 eV as a result of the interaction between the CMC and G. More insight into the HOMO/LUMO revealed that the properties of the HOMO/UMO have a major impact on the reactivity of molecules in chemical reactions. A molecule’s ability to donate electrons is represented by its HOMO; a higher HOMO energy level denotes a stronger tendency to donate electrons, increasing vulnerability to electrophilic attack. The LUMO, on the other hand, represents the electron acceptor potential; a lower LUMO energy level indicates a greater propensity to accept electrons, increasing the molecule’s susceptibility to nucleophilic attack. One important factor that influences the likelihood of a reaction is the energy difference between the HOMO and LUMO of two molecules; a smaller gap promotes electron transfer and, consequently, the occurrence of a reaction. It could be concluded here that the observed decrease in the HOMO/LUMO band gap confirmed the optical analysis.

Additionally, to confirm the increased responsibilities of CMC due to the reaction with G, MESP maps were calculated at the same theoretical level. The electron-rich and electron-poor reactive sites could be determined using the MESP. The MESP’s green area denotes a region that is almost neutral, while the red and blue regions indicate areas that are electron-rich and electron-poor, respectively. The MESP maps of the suggested structures of the CMC and CMC/G created with GaussView software at the same theoretical level are shown in Figure 8c,d, respectively. Red, orange, yellow, green, and blue are the order in which the potential drops [26,57]. The figures show that the electronic charges within the CMC model were redistributed and increased due to the interaction with the G as the red regions increased and extended within the whole structure of the CMC.

Moreover, to confirm the experimental FT-IR analysis, the IR frequencies were calculated theoretically at the same level. Figure 8e presents the IR spectrum of the CMC and CMC/G model molecules calculated using the B3LYP/6-31G (d, p) model. The theoretically determined IR and experimental FT-IR spectra of the CMC and CMC/G NCs were found to agree as well, as presented in Table 1 and Table 6. This confirms that the suggested structures of the CMC and CMC/G model molecules were valid and that the 6-31G (d, p) basis set was appropriate for IR frequency computations. It was discovered that these data matched the values published in the literature [16,17].

## 4. Conclusions

CMC/G nanocomposites were prepared with different weight percentages of G using a mechanical milling method. The structural behavior of the obtained samples was examined through SEM, FTIR, and XRD techniques.

The FTIR indicated the formation of CMC/G nanocomposites. The SEM images indicated that the CMC could be described as semi-crystalline with a flattened texture. A higher dispersion stability and less phase separation were observed with a high filler distribution of G particles within the polymer chains.

The CMC XRD peak profiles slightly changed due to the addition of G, which indicated that the degree of crystallinity of CMC was affected, which promoted crystallization in the CMC by facilitating intrachain conformational ordering. The crystallite size indicated that the incorporation of the G into the CMC significantly affected its crystallinity, which could then affect its overall properties.

The transmittance of the CMC/G nanocomposite at around 270 nm until reaching a value close to zero in the UV-C (100–280 nm) was observed. This was an indication that the CMC/G nanocomposite acted as a protective layer against UV-C.

Further optical analysis using the UV–Vis spectrophotometer was performed to determine a number of optical characteristics for both pristine CMC and CMC/G NCs, including absorbance, transmittance, opacity, absorption and extinction coefficients, optical band gap energy, and Urbach energy. The optical band gap energy and the Urbach energies of the CMC/G nanocomposites were evaluated using the Tauc model. The findings revealed that the crystallite size of the CMC decreased by approximately 50% of the magnitude compared with the CMC/G nanocomposite. The FT-IR analysis revealed the creation of hydrogen bonding of the CMC matrix and G particles. As the amount of G in the CMC composites increased, the nanocomposites’ optical band gap energies decreased. This result suggests that the G doping caused the nanocomposites to transition from an insulating stage to a semiconductor stage. The low values of the optical band gap energies were the consequence of the nanocomposites’ increased free carriers and inter-band localized energy states. The obtained low transmittance values for CMC films after adding small specific concentrations of G suggest that they can be used in various applications, such as food packaging, coatings, and active ingredients in skin care products.

Although CMC is a biodegradable and reactive biopolymer, DFT results indicated that the CMC/G nanocomposite is also reactive, as observed by its higher TDM values and lower HOMO/LUMO band gap. The MESP maps indicated that the electronic charges within the CMC model were redistributed and increased due to the interaction with G, which formed a reactive surface for the CMC/G nanocomposite. The calculated IR showed no negative frequencies, which is an indication of the optimal structure of the studied CMC and CMC/G models. All the calculated parameters indicated the reactivity of the CMC/G nanocomposite with the lowered band gap. It was concluded that this DFT study contributed the obtained results about the bond formation of hydrogen and the decreased band gap.

## Figures and Tables

**Table 1:** FT-IR bands of non-filled CMC and filled CMC/G nanocomposites.

Wavenumber/Sample							Band Assignment
CMC	CMC/0.2 wt.% G	CMC/0.4 wt.% G	CMC/0.8 wt.% G	CMC/1.0 wt.% G	CMC/2.0 wt.% G	CMC/3.0 wt.% G	
3292	3294	3294	3232	3282	3223	3231	OH stretching
2906	2908	2908	2900	2915	2871	2883	CH_2_ stretching
2850	2851	2851	2855	2856	2856	2856	CH_2_ asymmetric stretching
1584	1583	1583	1582	1582	1582	1581	Carboxylate group (COO–) asymmetric stretching
1411	1410	1410	1410	1409	1410	1410	CH_2_ scissoring
1319	1320	1320	1320	1320	1319	1319	OH bending
1049	1050	1050	1050	1051	1048	1049	C–O–C bending vibration
1017	1017	1017	1017	1017	1015	1015	C–O bond of the CH_2_OH group

**Table 2:** The crystallite size of CMC and CMC/G nanocomposites.

Sample	Crystallite Size (nm)
CMC	80.71
CMC/0.2 wt.% G	28.48
CMC/0.4 wt.% G	34.76
CMC/0.8 wt.% G	41.71
CMC/1.0 wt.% G	42.93
CMC/2.0 wt.% G	41.53
CMC/3.0 wt.% G	40.92

**Table 3:** UV transmittance percentage of CMC and CMC/G nanocomposite samples.

Sample	Transmittance (%)			Reference
	UV-C	UV-B	UV-A	
CMC	60	79	87	Current study
CMC/0.2 wt.% G	83	86	90	//
CMC/0.4 wt.% G	67	73	81	//
CMC/0.8 wt.% G	67	71	79	//
CMC/1.0 wt.% G	66	65	72	//
CMC/2.0 wt.% G	52	49	57	//
CMC/3.0 wt.% G	53	51	58	//
CMC/8% CuO	0	1	1	[16]
CMC/4% CuO@ZnO core/shell	0	0	0	[17]
CMC/lignin	-	2	11	[45]
PMMA/0.05% ZnO quantum dots	0	0	50	[46]
PVA/5% dopamine– melanin	0	0	30	[47]

**Table 4:** Indirect band gap and Urbach energy CMC and CMC/G nanocomposites.

Sample	$E_{g}^{in}$	E_U_ (eV)
CMC	5.27	0.34
CMC/0.2 wt.% G	5.30	0.45
CMC/0.4 wt.% G	5.24	0.55
CMC/0.8 wt.% G	5.16	0.57
CMC/1.0 wt.% G	5.13	0.68
CMC/2.0 wt.% G	4.90	0.81
CMC/3.0 wt.% G	4.81	0.94

**Table 5:** B3LYP/6-31G (d, p) calculated total dipole moment (TDM) and HOMO/UMO band gap energy (∆E) for both CMC and CMC/G nanocomposites.

Sample	TDM (Debye)	∆E (eV)
CMC	12.15	4.22
CMC/G	8.05	3.68

**Table 6:** B3LYP/6-31G (d, p) calculated IR band assignment of CMC and CMC/G models.

Wavenumber (cm^−1^)		Band Assignment
CMC Model	CMC/G Model	
3248	3239	OH stretching
2992	2985	CH_2_ stretching
2963	2945	CH_2_ asymmetric stretching
1641	1632	Carboxylate group (COO–) asymmetric stretching
1452	1455	CH_2_ scissoring
1344	1343	OH bending
1093	1096	C–O–C bending vibration
1013	1017	C–O bond of the CH_2_OH group

## Data Availability

The original contributions presented in this study are included in the article. Further inquiries can be directed to the corresponding author.
